# Improved high-throughput quantification of luminescent microplate assays using a common Western-blot imaging system

**DOI:** 10.1016/j.mex.2017.10.006

**Published:** 2017-10-27

**Authors:** Liam J. Hawkins, Kenneth B. Storey

**Affiliations:** Institute of Biochemistry and Department of Biology, Carleton University, 1125 Colonel By Drive, Ottawa, Ontario K1S 5B6, Canada

**Keywords:** Improved luminescent assay quantification using a Western-blot imaging system, Luminescent assay quantification, HDAC activity, Epigenetic assay

## Abstract

Common Western-blot imaging systems have previously been adapted to measure signals from luminescent microplate assays. This can be a cost saving measure as Western-blot imaging systems are common laboratory equipment and could substitute a dedicated luminometer if one is not otherwise available. One previously unrecognized limitation is that the signals captured by the cameras in these systems are not equal for all wells. Signals are dependent on the angle of incidence to the camera, and thus the location of the well on the microplate. Here we show that:

• The position of a well on a microplate significantly affects the signal captured by a common Western-blot imaging system from a luminescent assay.
• The effect of well position can easily be corrected for.
• This method can be applied to commercially available luminescent assays, allowing for high-throughput quantification of a wide range of biological processes and biochemical reactions.

## Graphical abstract

**Figure:**
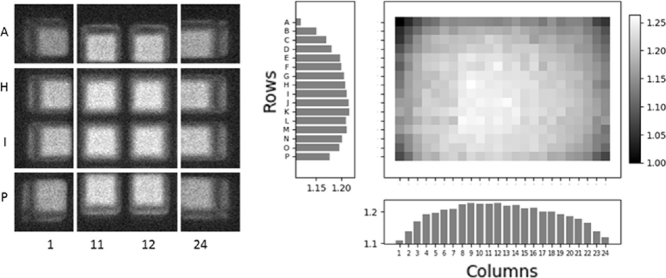

## Method details

### Background information

**Fig. 1:**
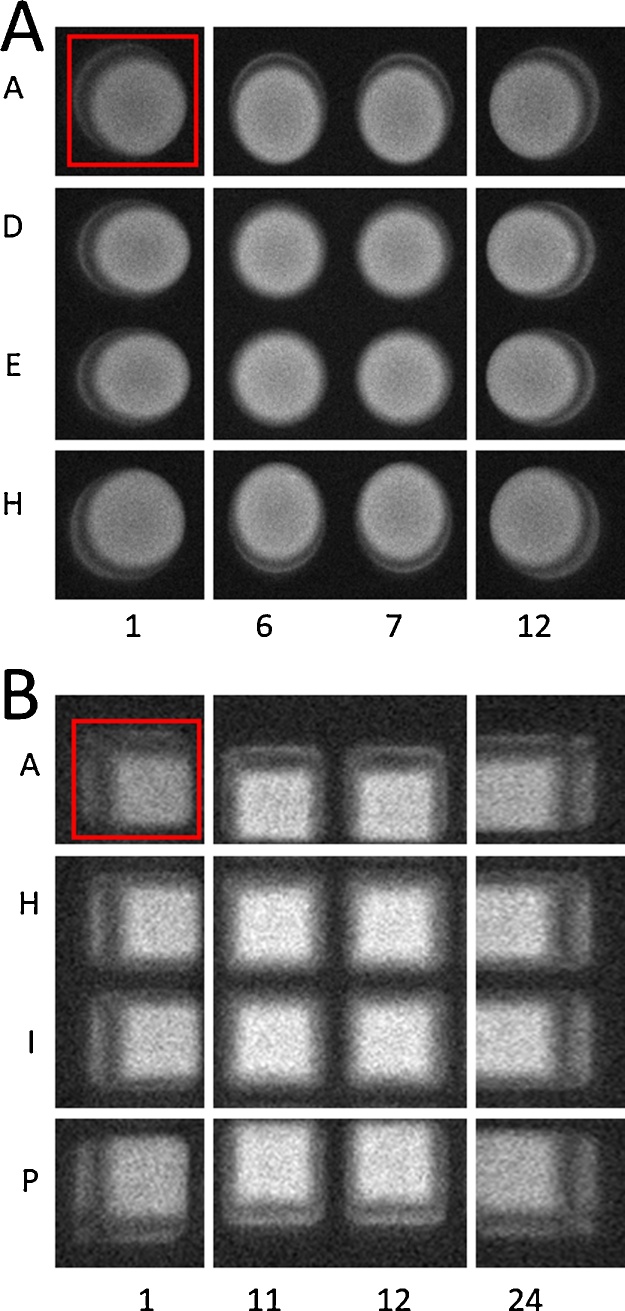
Demonstration of non-uniform view of wells from acquisition software. (A) Segmented view of 96-well black microplate. Each well contains a 2:1 v:v mixture of chemiluminescent solution and HRP-conjugated anti-rabbit antibody (1:8000 v:v in TBST) for a final volume of 100 μL. (B) Segmented view of 384-well black microplate. Each well contains a 2:1 v:v mixture of chemiluminescent solution and HRP-conjugated anti-rabbit antibody (1:8000 v:v in TBST) for a final volume of 40 μL. Well-position effect is most apparent when comparing center wells to edge or corner wells. Red boxes are examples of area selected for measurement in quantification software.

Luminescent assays offer a simple, sensitive, and high-throughput method for measuring a vast range of biological processes. Since the emitted light from luminescent assays is the result of a chemical or biochemical reaction, it is uniquely less complex than other assay detection modalities such as absorbance or fluorescence. These methods require equipment that at a minimum contains a light source and sensor, while luminescent detection requires just a sensor. Whether a luciferase based reporter assay is used to study gene expression, or chemiluminescent assay for measuring biochemical reactions, it is generally thought that a spectrophotometer capable of detecting the luminescence signal is required. Previous reports have demonstrated that common inexpensive devices, such as smartphones, can replace expensive conventional equipment [1], [2]. The simplicity of measuring luminescence can be taken advantage of since other types of equipment, such as CCD cameras, can convert light emitted from a luminescent reaction to a digital signal for quantification. Previously it was shown that a Western-blot imaging system can be adapted to reliably detect and quantify luminescent signals [3]. Expanding the use of a common piece of laboratory equipment such as this can be preferable to purchasing a dedicated luminometer, if one is not already present. Although this technique has comparable performance to a dedicated luminometer, an additional source of error is introduced when measuring a high number of microplate wells, or if the measured wells are far apart. As demonstrated in Fig. 1, the appearance to the camera of a microplate well is dependent on the angle of incidence to the camera itself. Wells located close to the optical axis of the lens show a complete view of the contents of the well, whereas the contents of wells further from the optical axis are partially obstructed on one side of the well and signal is reflected off the wall of the opposite side. This perspective error, which we refer to as well position effect, is due to the entocentric lens used in conventional cameras. This well position effect could be eliminated optically by replacing the entocentric lens with a telecentric lens. Unlike entocentric lenses, with telecentric lenses the chief rays from an object are parallel to the optical axis of the lens, meaning magnification and perspective is consistent regardless of the objects perpendicular distance to the optical axis. Telecentric lenses have been used for biological imaging and sensing applications previously [4], [5], however, they are significantly more complex and expensive than entocentric lenses, and thus would mitigate any of the cost savings generated by using a Western-blot imaging system instead of purchasing a luminometer. Here we present an improved method of luminescence assay quantification using a common, un-modified, Western-blot imaging system that corrects for this well position effect, increasing precision of measurements across a microplate.

### Equipment setup

**Fig. 2:**
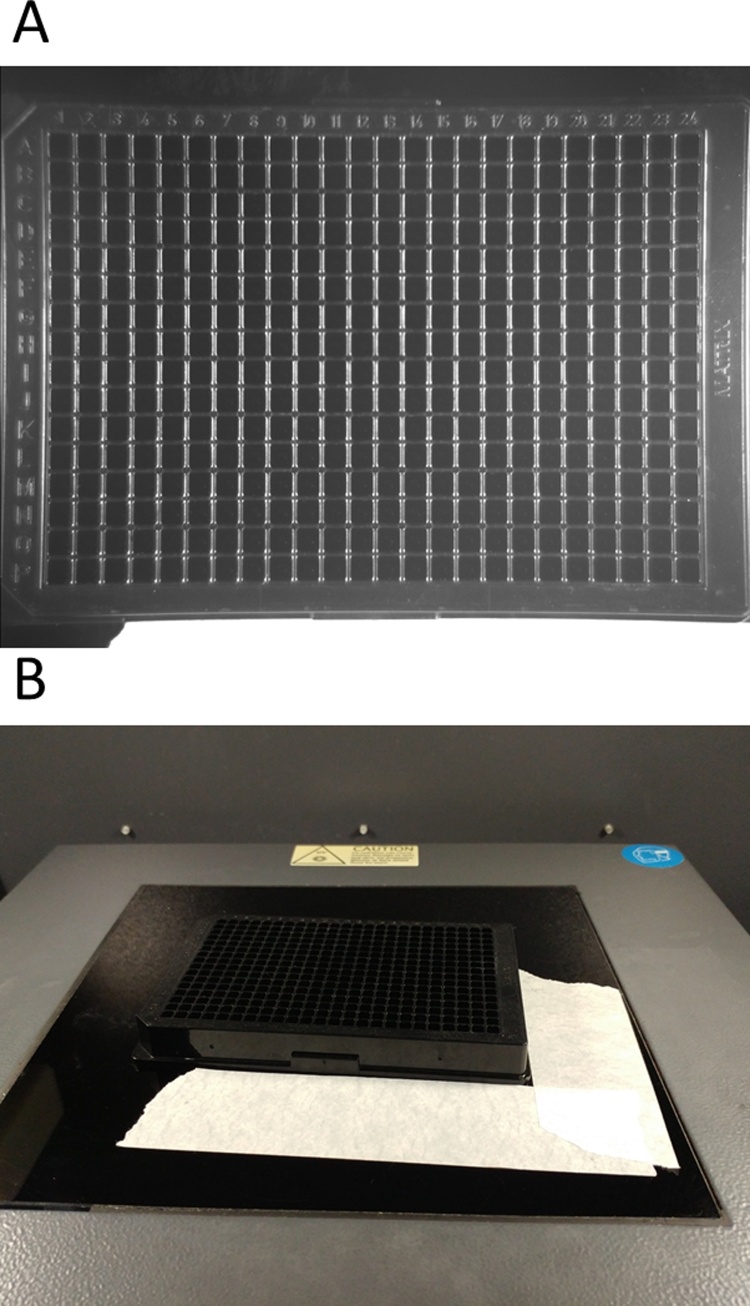
Alignment of 384-well microplate in Western-blot imaging system. (A) The microplate is aligned on the stage in a Syngene G:BOX so that all wells are visible in the acquisition software. (B) Masking tape is placed around one corner for reproducible alignment after the microplate is been taken out of the system.

A 96- or 384-well Costar opaque black microplate was placed on the platform of a western-blot imaging system (G:BOX, Syngene, Frederick, MD). Using the live acquisition software, the microplate was aligned to the center of the view of the camera so all wells were visible (Fig. 2A). Masking tape was placed around one corner of the microplate to act as an alignment guide for subsequent readings (Fig. 2B). Aperture size, zoom, and focus settings from the acquisition software were recorded and kept constant for all experiments.

### Well scaling factor determination

**Fig. 3:**
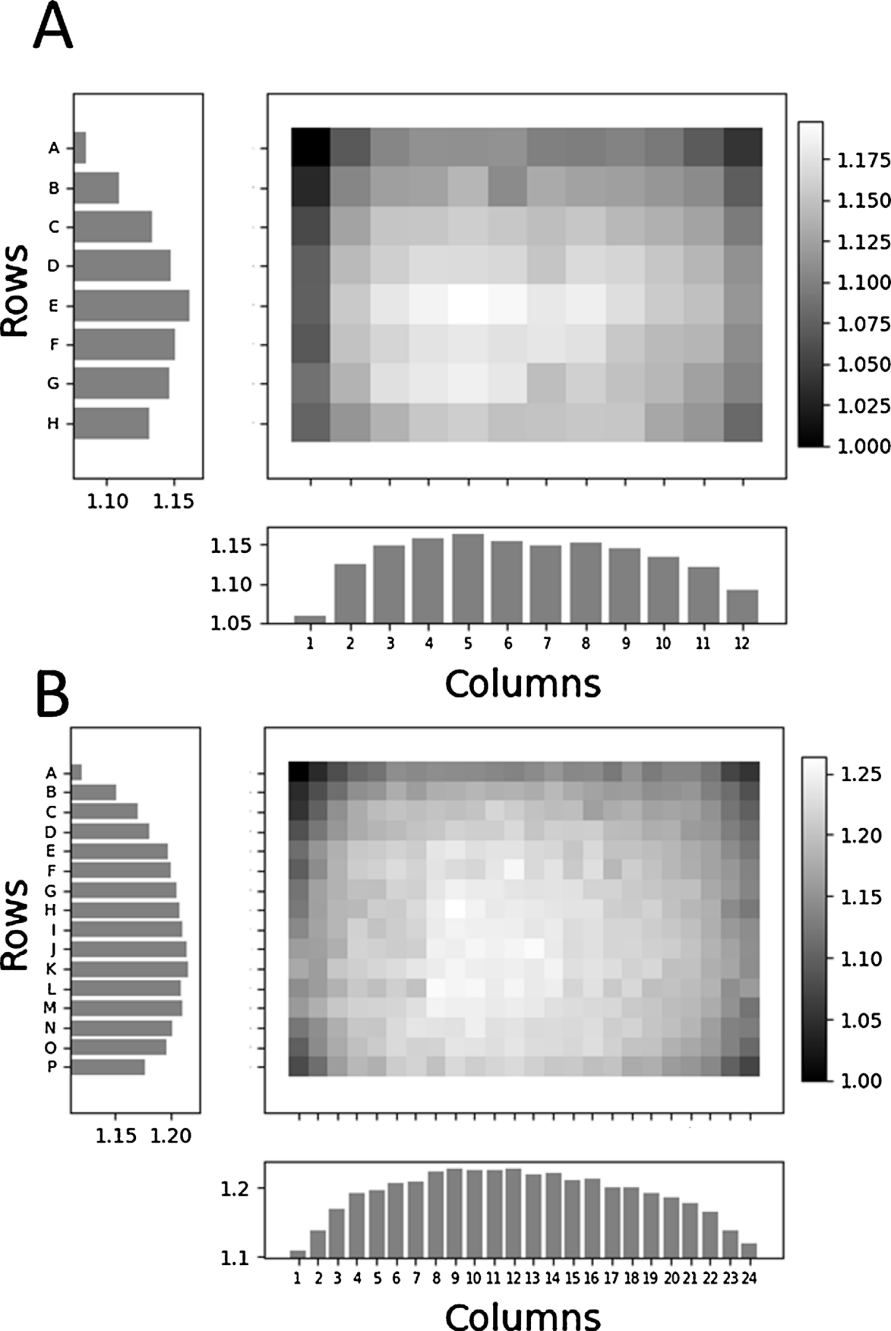
Well scaling factors for (A) 96- and (B) 384-well microplates. Scaling factors are normalized to luminescence of well A1 (upper-left). Lighter shading on heatmap indicates wells with higher well scaling factors. Left bars are average scaling factor of each corresponding row of wells, bottom bars are average scaling factor of each corresponding column of wells.

A 2:1 v:v mixture of chemiluminescent solution (1.25 mM luminol, 200 μM p-coumaric acid, 1:3000 v:v H_2_O_2_, and 100 mM tris buffer pH 8.8) and HRP-conjugated anti-rabbit antibody (1:8000 v:v in 0.05% TBST) was pre-mixed, then added to every well for a total volume of 100 μL in the 96-well microplates or 40 μL in the 384-well microplates. These volumes were chosen because they are within the range of working volumes recommended by the manufacturer (75–200 μL for 96-wells and 20–80 μL for 384-wells) and match the volume used in the subsequent assay. The plate was then briefly shaken and placed inside the imaging system using the masking tape for alignment where it sat for 5 min to allow for signal stabilization. An image was captured with a 2 min exposure time, and the accompanying GeneTools software was used to measure the signal of each well. The 2 min exposure time was chosen because it maximized the number of serial dilutions that could be detected in the linear range, without over exposing the higher luminescent wells. In the GeneTools quantification software, each well was captured in its entirety (red boxes, Fig. 1) and the densitometric raw volume determined by GeneTools was used as a measure of signal intensity. Every wells signal intensity was normalized to well A1 (upper-left) of its respective plate, resulting in a scaling factor for each well. Scaling factors are visualized in Fig. 3 where it is demonstrated that wells positioned centrally have higher scaling factors than wells positioned peripherally due to their lower angle of incidence. Plate format affects the magnitude of the well position effect, signified by the fact that 384-well microplates have a higher maximum scaling factor than 96-well microplate (Fig. 3). This is due to the necessary changes in well geometry as more wells are fitted into the same microplate footprint. Wells from 384-well microplates have a larger height-width ratio than 96-well microplates, and therefore a higher angle of incidence will cause a larger proportion of the wells contents to be obstructed by the well wall. We also successfully determined well scaling factors for volumes spanning the entire range of recommended working volumes for each plate (75, 110, 150, and 200 μL for 96-wells; 20, 40, 60, 80 μL for 384-wells) and found that the well position effect had a greater magnitude at lower relative volumes.

### Validating well scaling factor utility

To validate the utility of correcting for well position, HRP standard curves were run in duplicate on 96- and 384-well microplates. One set of standard curve replicates were placed in the upper-left corner (beginning with well A1, running horizontally) while the other was centered on the plate (beginning with well D3 for 96- or well H9 for 384-well microplates, running horizontally) with the intent of maximizing the well position effect. A 2:1 v:v mixture of chemiluminescent solution and varying concentrations of HRP-conjugated anti-rabbit antibody were added to each standard curve well for a total volume of 100 μL in the 96-well microplate and 40 μL in the 384-well microplate. The plate was then briefly shaken and placed in the imaging system where it sat for 5 min. Luminescence was determined as stated above for well scaling factor determination.

**Fig. 4:**
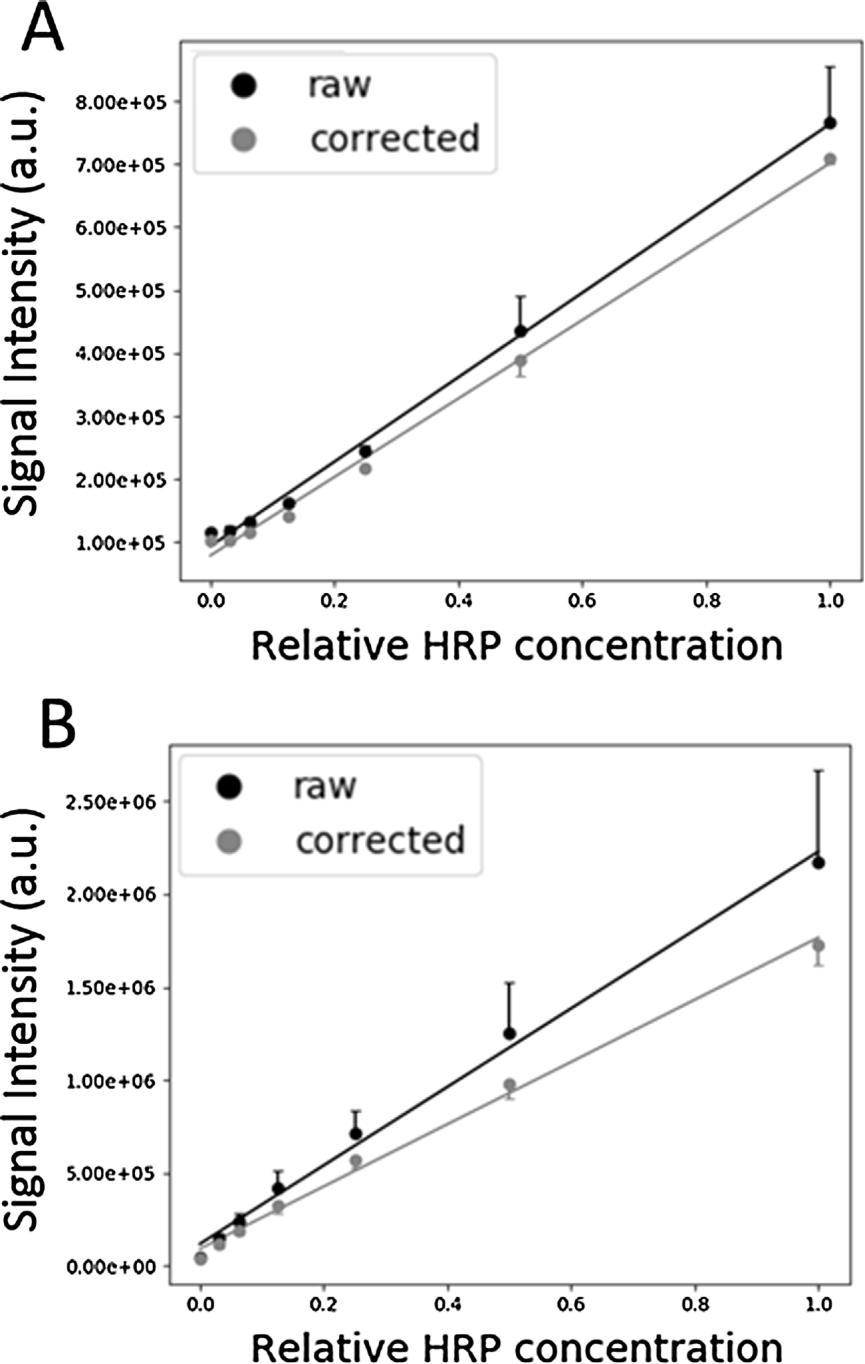
HRP standard curves before and after well normalization. Non-corrected (raw) and corrected standard curves for (A) 96- and (B) 384-well microplates. Standard curves were run in duplicate, one replicate starting in the top left well and the other central of each microplate, as to maximize well-position effect. A 2:1 v:v mixture of chemiluminescent solution and varying concentrations of HRP-conjugated anti-rabbit antibody were added to each standard curve well. Bottom errors bars for the raw series and top error bars for the corrected series are omitted for visual clarity. R^2^ values are found in Table 1. a.u = arbitrary units.

To correct for well position effect, the measured luminescence of each well was divided by its corresponding well scaling factor as previously determined. Raw and corrected HRP standard curves are plotted for 96- and 384-well microplates in Fig. 4A and B respectively. With little to no change in R^2^ values between raw and corrected standard curves, the average standard deviation between duplicates decreased by 380.1% and 399.1% for 96- and 384-well microplates respectively (Table 1). Given that the ground-truth values of the replicates should be equal (with some pipetting error), the large standard deviations from the raw standard curves is due to well position effect, which is subsequently minimized when corrected for.

**Table 1:** Results from HRP standard curves for 96- and 384-wells microplates, before and after well-position correction.

Microplate format	Values	R^2^	Average SD (AU)	ΔSD after correction (%)
96-well	Raw	0.997	4.01 × 10^4^	−380.1
	Corrected	0.996	1.05 × 10^4^	
384-well	Raw	0.994	2.23 × 10^5^	−399.1
	Corrected	0.994	5.58 × 10^4^	

Abbreviations: R^2^ = Coefficient of determination, SD = Standard deviation, AU = Arbitrary units.

### Application to HDAC-Glo I/II assay

**Fig. 5:**
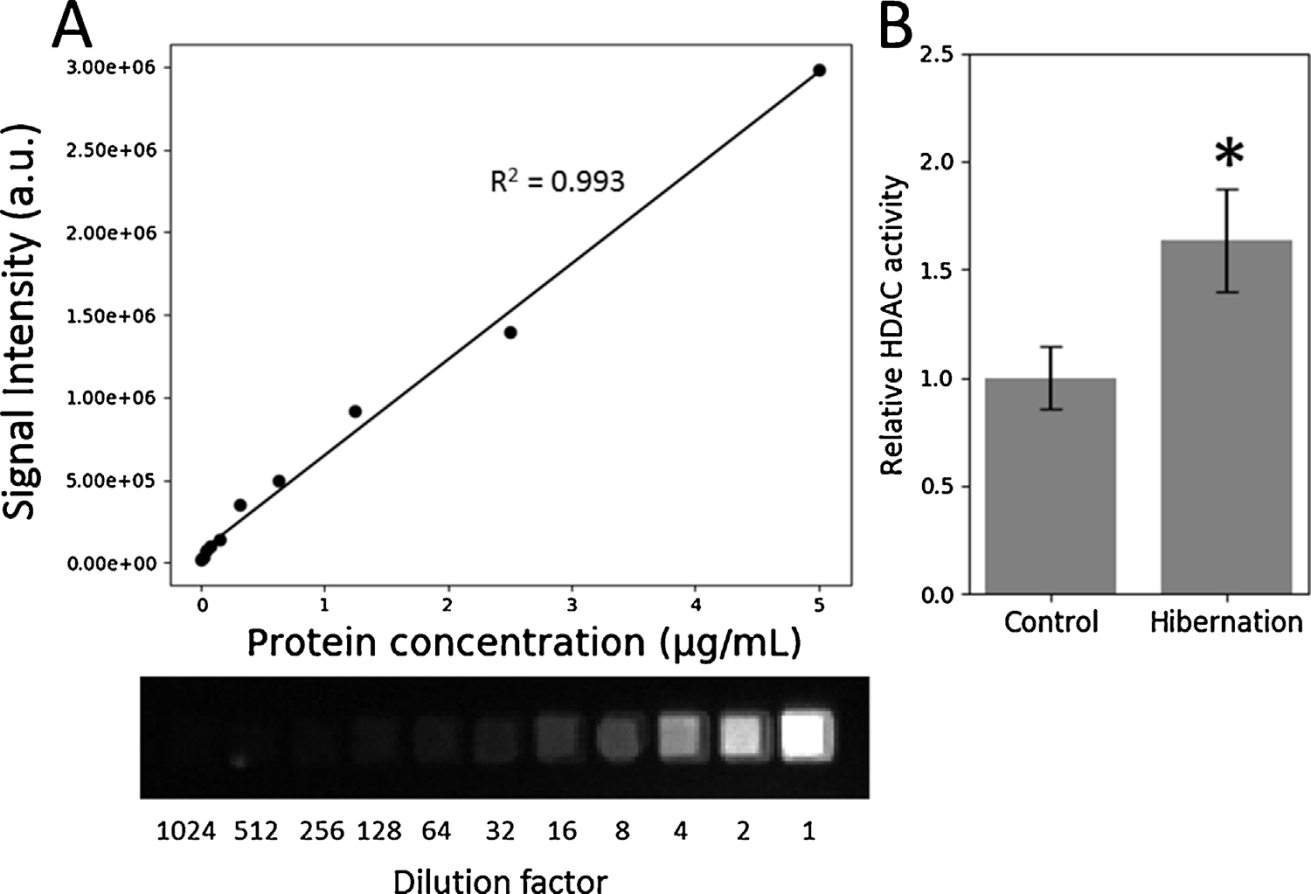
Well position effect correction applied to commercially available luminescent HDAC-Glo I/II activity assay. (A) Protein standard curve for the HDAC-Glo I/II assay using 13-lined ground squirrel skeletal muscle total protein extracts after well position effect correction. Beneath the standard curve is the sequence of luminescent wells captures with a Syngene G:BOX and used to generate the standard curve. The standard curve was performed in wells B1-B11. (B) Relative HDAC class I/II deacetylase activity in skeletal muscle of 13-lined ground squirrel during euthermic control and hibernation with well position effect correction. Samples were measured in wells D1-K1 Data are mean ± SEM, *n* = 4 for each condition. * indicates statistical significance (*p* < 0.05). a.u = arbitrary units.

The relative activity of histone deacetylase (HDAC) class I and II enzymes from skeletal muscle was determined using the commercially available HDAC-Glo I/II assay (Promega, Catalogue #G6420) with the above method, including well position effect corrections. Total soluble protein extracts were prepared from skeletal muscle of control and hibernating 13-lined ground squirrel, *Ictidomys tridecemlineatus* (Animals were treated as in [6]. See Appendix A for total soluble protein extraction protocol). The linear dynamic range of the assay was first determined as per the manufacturer’s instructions. Briefly, the HDAC-Glo I/II reagent was prepared by rehydrating the HDAC-Glo I/II substrate (Boc-GAK(Ac)-aminoluciferin) in 10 mL of HDAC-Glo I/II assay buffer (25 mM Tris buffer pH 8.0, 137 mM NaCl, 2.7 mM KCl, 1 mM MgCl_2_, and 1% v/v Triton X-100) with 10 μL of supplied developer reagent. Serial twofold dilutions of pooled total protein extracts (starting at 5 μg/mL, and including 0 μg/mL) were prepared by diluting protein extracts with HDAC-Glo I/II assay buffer. Equal volumes of protein dilutions and HDAC-Glo I/II reagent were added to the wells of a 384-well Costar opaque black microplate for a final volume of 40 μL, which was then briefly shaken and incubated at room temperature for 15 min. Luminescence was determined as above and values were divided by previously determined corresponding well-scaling factors (Fig. 5A). A higher signal corresponds to higher HDAC class I/II enzyme activity. The HDAC-Glo I/II substrate (Boc-GAK(Ac)-aminoluciferin) present in the HDAC-Glo I/II reagent has an acetyl-group that is susceptible to removal by HDAC class I and II enzymes. When the acetyl-group is removed, the deacetylated substrate (Boc-GAK-aminoluciferin) becomes sensitive to proteolytic enzymes in the developer reagent, producing aminoluciferin. Luciferase also present in the developer reagent oxidizes aminoluciferin and produces the luminescent signal [7].

Control (n = 4) and hibernation (n = 4) total protein extracts were each diluted in HDAC-Glo I/II assay buffer to 1.25 μg/mL, and were added in equal volume with HDAC-Glo I/II reagent to the microplate for a final volume of 40 μL. The plate was briefly shaken, incubated for 15 min at room temperature, and luminescence was determined as above including correction for well-position effect (Fig. 5B).

## Additional information

DNA coils around histone proteins to form a nucleosome, the core structural unit of a chromosome. Chemical modifications of these histone proteins contribute to how tightly packed the DNA is, and thus are used as regulatory mechanisms for controlling access to the DNA (for review see Ref. [8]). The tighter the DNA is packed, the harder it is for the transcriptional machinery to access the DNA, thus decreasing transcription compared to the more loosely packed DNA. Perhaps one of the most well characterized histone modification is histone acetylation. When histones are acetylated, the histone-DNA interaction is weakened and causes the DNA to be packed more loosely, and thus becoming more transcriptionally permissive [9]. Histone deacetylases (HDACs) are enzymes that catalyze the removal of acetyl-groups from histone proteins, and have long been known to be transcriptional repressors [10].

The 13-lined ground squirrel (*I. tridecemlineatus*) is a hibernating mammal that drastically reduces its metabolic rate during the winter months to survive when food resources are scarce. While in deep torpor, body temperature, heart rate, and breathing rate decrease significantly, resulting in a metabolic expenditure that is 1–5% of euthermic animals [11]. There is evidence that this reduction in metabolic rate is accompanied by a global decrease in transcriptional output [6], [12], [13], which would be beneficial to the animal as energy resources would not be wasted on the overproduction of unnecessary proteins. A likely contributor to the global decrease in transcription during hibernation is the increase that has been shown in HDAC class I and II enzymes and corresponding decrease in their target acetyl-histone residues [6]. Both HDAC1 and HDAC4 increased approximately 1.2 and 1.4-fold respectively, while an approximate 40% decrease in H3K23ac was seen in animals during hibernation [6]. By using the HDAC-Glo I/II assay with the method described above, we show here that the enzymatic activity of HDAC class I and II enzymes do in fact increase during hibernation (Fig. 5B), which aligns with those results, adding to the evidence that HDACs contribute to a decreased transcriptional state during hibernation.

The improvements we have made allows for high-throughput applications of luminescent assays in multiple microplate formats that may otherwise be impossible with a standard luminometer. For example, most luminometers are restricted to a single microplate format (commonly 96-wells), and therefore do not allow for higher-throughput formats such as 384-well. Additionally, since all wells are measured simultaneously with this method, it may be possible to perform experiments that rely on a series of measurements over time, such as enzyme kinetics, with precision that would be greater than using a luminometer that measures wells in sequence.

While this method pertained only to the detection of visible light from luminescent assays using the Syngene G:BOX, it should be noted that this method could be applied to other detection modalities. Many Western Blot imaging systems are equipped with UV and fluorescent excitation and detection capabilities, and thus assays using these wavelengths could be measured in a similar manner. This also opens the possibility for multiplexed assays. For example, separate fluorophore-conjugated antibodies could be used to measure multiple targets in a single microplate well.

The sensitivity of this method is a function of the hardware, software, and assay used. Sensitivity was not a problem for our purposes, as demonstrated in Fig. 5A, we were able to detect a linear signal down to a dilution factor of 1024, corresponding to approximately 200 pg of protein extract. If needed, the sensitivity could be increased further by increasing the exposure time, or increasing pixel-binning. We chose to use black microplates to stay consistent with previous methods [3], however white microplates are often recommended for luminescent assay as they reflect light more efficiently, resulting in greater sensitivity.

Quantification of image-based optical signals, as shown here, is a common technique, particularly in Western blot analysis. The conversion of light, to a signal processed by the camera, to pixel intensities, to volumetric quantification in software, is not problem free. Specific to densitometric analysis of Western blots, attempts have been made to identify errors and establish frameworks for standardized quantification [14], [15], [16]. The issues identified in these articles can be applied broadly to other application such as the method we have outlined here. Determining the limits of detection and measurement linearity are two aspects that are easiest to overlook, and yet can have a large effect on the data produced.

Using this method does take significantly more time than a luminometer since a separate plate must be set-up to correct for well position. Quantification of both the calibration and experimental plates take more time, compared to most automated luminometers, since you must tell the software the size and position of each well. Although there are additional time and reagent requirements, the use of a common piece of equipment such as a Western Blot imaging system to measure luminescent signals opens the doors for researchers to perform assays in microplate formats without the need for dedicated luminometers.

## Appendix A

### Total soluble protein extraction

A mortar and pestle was pre-cooled with liquid N_2_, and skeletal muscle samples were weighed, crushed, and then homogenized 1:2 w/v in pre-chilled homogenization buffer (20 mM HEPES, 200 mM NaCl, 0.1 mM EDTA, 10 mM NaF, 1 mM Na_3_VO_4_, 10 mM, 10 mM β-glycerophosphate, 1 μL/mL protease inhibitor [BioShop Canada Inc., Catalogue #PIC002], and a few crystals of PMSF, pH 7.4). The homogenates were centrifuged at 4 °C at 10,000 × *g* for 15 min. The supernatant was collected, and protein concentration was determined using the BioRad protein assay (BioRad Laboratories, Catalogue #5000002). Sample concentrations were adjusted to 5 μg/μL using the above homogenization buffer. Samples were then stored at −80 °C until use.
